# mTOR Signaling: The Interface Linking Cellular Metabolism and Hepatitis B Virus Replication

**DOI:** 10.1007/s12250-021-00450-3

**Published:** 2021-09-28

**Authors:** Xueyu Wang, Zhiqiang Wei, Yongfang Jiang, Zhongji Meng, Mengji Lu

**Affiliations:** 1 Department of Infectious Diseases, The Second Xiangya Hospital, Central South University, Changsha, 410011 China; 2 Institute of Virology, University Hospital Essen, University of Duisburg-Essen, 45122 Essen, Germany; 3 Institute of Biomedical Research, Hubei Clinical Research Center for Precise Diagnosis and Treatment of Liver Cancer, Taihe Hospital, Hubei University of Medicine, Shiyan, 442000 China; 4 Department of Infectious Diseases, Taihe Hospital, Hubei University of Medicine, Shiyan, 442000 China

**Keywords:** Mammalian target of rapamycin (mTOR), Hepatitis B virus (HBV), Metabolism, Autophagy

## Abstract

Mammalian target of rapamycin (mTOR) is a conserved Ser/Thr kinase that includes mTOR complex (mTORC) 1 and mTORC2. The mTOR pathway is activated in viral hepatitis, including hepatitis B virus (HBV) infection-induced hepatitis. Currently, chronic HBV infection remains one of the most serious public health issues worldwide. The unavailability of effective therapeutic strategies for HBV suggests that clarification of the pathogenesis of HBV infection is urgently required. Increasing evidence has shown that HBV infection can activate the mTOR pathway, indicating that HBV utilizes or hijacks the mTOR pathway to benefit its own replication. Therefore, the mTOR signaling pathway might be a crucial target for controlling HBV infection. Here, we summarize and discuss the latest findings from model biology research regarding the interaction between the mTOR signaling pathway and HBV replication.

## Introduction

Hepatitis B virus (HBV) infection remains a global threat to public health. Patients with chronic HBV infection are prone to cirrhosis, liver failure, and hepatocellular carcinoma (HCC) (Tang *et al.* 2018). In the past few decades, effective vaccines have been widely used to reduce the incidence of HBV infection, especially in newborns. However, the current therapeutic strategies for HBV infection using pegylated interferon and nucleos(t)ide analogues have limited efficiency against the disease (Liang *et al.* 2015; Subic and Zoulim 2018; Fanning *et al.* 2019).

HBV, which belongs to the *Hepadnaviridae* family, is an enveloped virus with a double-stranded DNA genome of a size of 3.2 kb, and is thus one of the smallest DNA viruses currently known (Karayiannis 2017). HBV replicates through reverse transcription (Glebe and Bremer 2013; Tong and Revill 2016). HBV covalently closed circular (ccc) DNA copies are formed in the nucleus, then serve as templates for viral transcription, and generate five major RNA molecules. These molecules include 3.5-kb pregenomic (pg) RNA and preC RNA, 2.4- and 2.1-kb preS/S mRNAs, and 0.7-kb HBx mRNA, which encode core/polymerase proteins, precore protein, large envelope protein, middle and small envelope proteins, and X protein, respectively (Gish *et al.* 2015; Nassal 2015). Subsequently, the core protein, pgRNA, and viral DNA polymerase are recruited to form nucleocapsids (NCs). In the process of virion release, NCs can directly be assembled with glycoprotein envelope and trigger the secretion of nascent virions, or be reintroduced into the nucleus for amplification of the cccDNA pool (Guo *et al.* 2007a; Kock *et al.* 2010). Noninfectious subviral particles (SVPs) composed of three envelope proteins, S-, M-, and L- HBV surface antigen (HBsAg), are independently secreted from host cells only through the endoplasmic reticulum (ER)-Golgi secretion pathway.

The life cycles of many viruses can be regulated by the mammalian target of rapamycin (mTOR) signaling pathway (Ranadheera *et al.* 2018; Bossler *et al.* 2019). The mTOR signaling pathway is a key regulator of many cellular processes including cell metabolism, growth, proliferation, survival and immunity (Laplante and Sabatini 2012; Liu Y *et al.* 2015; Saxton and Sabatini 2017), and is abnormally overactivated in numerous human cancers (Ciuffreda *et al.* 2010; Forbes *et al.* 2011). Moreover, the mTOR signaling pathway is activated during infection by many viruses, including HBV. Previous studies have shown that the interaction of mTOR signaling pathway and HBV life cycle is complex. While HBV proteins like the HBV X (HBx) protein are able to modulate this pathway (Zhu *et al.* 2017; Wang X *et* *al*. 2019). HBV replication and gene expression are also regulated by the mTOR signaling pathway (Guo *et al. *2007b; Zhang *et al.* 2011; Huang *et al.* 2014; Li *et al.* 2015; Lin *et al.* 2017; Wang *et al.* 2020a, 2020b). In addition, the mTOR signaling pathway restricts or degrades viral particles and SVPs in a lysosome-dependent manner (Lin *et al.* 2019c; Wang *et al.* 2020b). In this review, we summarize and discuss the major findings in this field.

## mTOR Signaling Pathway

The mTOR protein is an evolutionarily conserved Ser/Thr kinase in the phosphoinositide 3-kinases (PI3K)-related kinase (PIKK) family. mTOR is the catalytic subunit of two complexes, mTORC1 and mTORC2 (Wullschleger *et al.* 2006); the localization of these complexes in different subcellular compartments affects their activation and function (Betz and Hall 2013).

mTORC1 is a rapamycin-sensitive companion of mTOR that acts as a sensor for growth factors, pressure, energy status, oxygen, and amino acids to control many major cellular processes, including glucose homeostasis, lipid synthesis, and autophagy (Fang *et al.* 2001; Kim *et al.* 2002; Hay and Sonenberg 2004). The heterodimer composed of tuberous sclerosis 1 (TSC1) and 2 (TSC2) is a key upstream regulator of mTORC1. The GTP-bound form of Ras homolog enriched in brain (Rheb) directly interacts with mTORC1 and strongly stimulates its kinase activity. However, TSC1/2 negatively regulates mTORC1 activity by reversing Rheb into its inactive GDP-bound state (Inoki *et al.* 2003a; Tee *et al.* 2003). Many upstream signals including growth factors that regulate mTORC1 through the PI3K and Ras pathways, are transmitted through the TSC1/2 complex. Effector kinases, including protein kinase B (PKB/Akt) (Chen *et al.* 2018), extracellular signal-regulated kinase 1/2 (ERK1/2) (Ma *et al.* 2005), and p90 ribosomal S6 kinase (RSK) (Roux *et al.* 2004), can directly phosphorylate and inactivate the TSC1/2 complex, thereby activating mTORC1. Amino acids also activate mTORC1. Under conditions with sufficient amino acid supply, mTORC1 is activated via localization with the Ragulator-Rag complex (RagA/B and RagC/D) on the lysosome surface after activating Rheb (Efeyan *et al.* 2012; Groenewoud and Zwartkruis 2013). The activation of mTORC1 can enhance protein or lipid synthesis by modulating the translation regulator p70 ribosomal S6 kinase 1 (S6K1) and the eukaryotic translation initiation factor 4E binding protein 1 (4EBP1) or sterol regulatory element-binding protein 1/2 (SREBP1/2) (Duvel *et al.* 2010; Wang *et al.* 2011; Guo *et al.* 2019), respectively. Han *et al.* proved that mTORC1 also regulates the trafficking and maturation of SREBP1 through CREB-regulated transcription coactivator 2 (CRTC2). Under nutrient-rich conditions or insulin stimulation, mTOR activation leads to the phosphorylation and activation of SREBP1, thereby enhancing lipogenesis (Han *et al.* 2015).

The signaling pathway triggered by mTOR competes with other regulators of cellular metabolisms, such as AMP-activated protein kinase (AMPK), another major metabolic sensor. AMPK is activated in response to the increased AMP/ATP ratio, for example, after starvation of glucose. Importantly, AMPK directly or indirectly inhibits mTOR activity (Inoki *et al.* 2003b; Gwinn *et al.* 2008). Mitochondria biogenesis and turnover mediated by mTOR and AMPK are essential for regulating the cellular metabolism, especially mitochondria. Peroxisome proliferator-activated receptor (PPAR) gamma coactivator 1α (PGC1α) is a nuclear cofactor that plays a key role in mitochondrial biogenesis, oxidative metabolism, and gluconeogenesis. A report showed that nuclear mTORC1 controls the transcriptional activity of PGC1α by changing its physical interaction with another transcription factor Yin Yang 1 (YY1) in skeletal muscle (Cunningham *et al.* 2007). However, AMPK directly phosphorylates and activates PGC1α in skeletal muscle (Jager *et al.* 2007). Additionally, under glucose starvation conditions, inactivation of mTORC1 is accompanied with increased expression of PGC1α in hepatoma cells (Wang *et al.* 2020a). Consistent with this finding, mTORC1 reduces the fasting-induced activation of PPARα (Sengupta *et al.* 2010).

In addition, mTORC1 enhances glycolysis and glucose uptake by regulating the transcription factor hypoxia-inducible factor (HIF1) (Semenza 2010) and promotes cell growth by negatively regulating autophagy, which is necessary for the recycling of damaged organelles and the adaptation of organisms to nutrient starvation (Kim *et al.* 2011). In mammals, mTORC1 directly phosphorylates and inhibits the ULK1/Atg13/FIP200 complex, which is an essential kinase complex that initiates autophagy. Activating mTORC1 can inhibit lysosome function by inhibiting the activity of transcription factor EB (TFEB), the master regulator of lysosomal biogenesis. Nutritional starvation or inhibition of mTORC1 activates TFEB by promoting its nuclear translocation, thereby promoting autophagic flux (Zhou *et al.* 2013).

On the other hand, mTORC2 is a rapamycin-insensitive companion of mTOR (Jacinto *et al.* 2004; Sarbassov *et al.* 2004) and can be directly activated by PI3K (Shimobayashi and Hall 2014). Interestingly, long-term rapamycin treatment can also inhibit mTORC2 in some cell types, which may be caused by inhibition of mTORC1 and reduction in binding of mTORC1 to the nascent mTORC2 complex (Delgoffe *et al.* 2011). Compared with the mTORC1 pathway, the mTORC2 pathway is much less understood. The downstream genes of mTORC2 include several members of the AGC kinase subfamily, such as *Akt*, serum and glucocorticoid-induced protein kinase 1 (S*GK1*) and protein kinase C-α (*PKC-α*). mTORC2 directly activates Akt by phosphorylating the hydrophobic motif (Ser473) of Akt, which is required for the maximum activation of Akt (Sarbassov *et al.* 2005). Akt regulates cellular metabolism, survival, apoptosis, growth and proliferation through phosphorylation of several effectors.

## HBV-Mediated Activation of mTOR Signaling Pathway

**Fig. 1:**
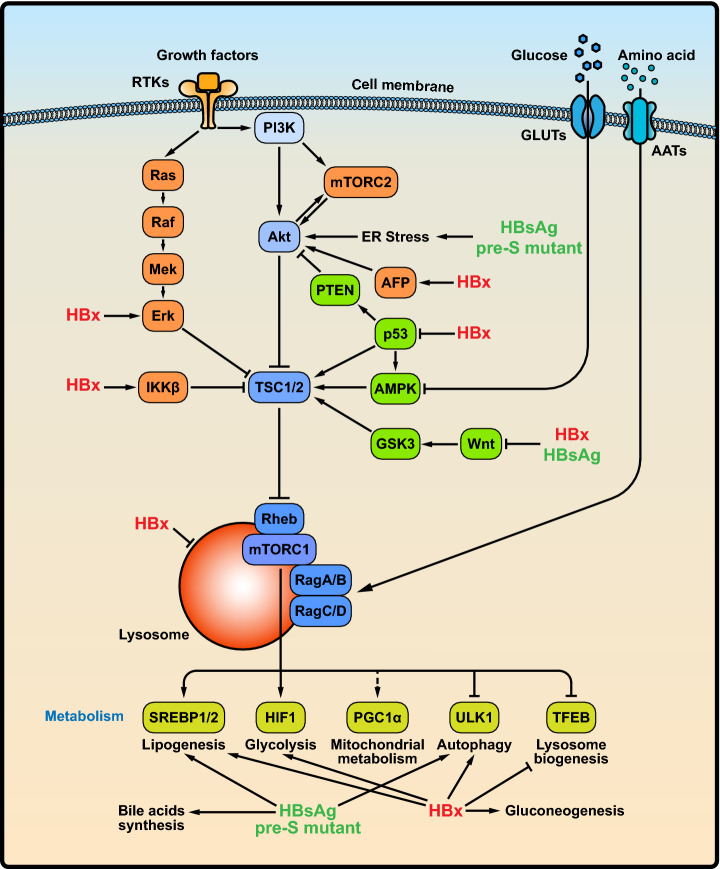
HBV infection activates the PI3K/Akt/mTOR signaling pathway. Growth factors and nutrition, including glucose and amino acids, are factors known to activate the PI3K/Akt/mTOR signaling pathway. During the HBV infection process, HBsAg and pre-S mutant protein may accumulate in the ER lumen to trigger ER stress, thereby activating the PI3K/Akt/mTOR signaling pathway. In addition, HBsAg and HBx indirectly activates the mTOR signaling by regulating Wnt/β-GSK3 pathway. HBx-mediated activation of the RAS/RAF/MAPK pathway and IKKβ induces the mTOR signaling pathway by inhibiting the activity of TSC1/2. Additionally, HBx indirectly modulates p53, AFP, or Wnt/β-GSK3 pathway to activate the mTOR signaling pathway. More importantly, HBV infection interferes with hepatic metabolism signaling pathways, including glucose homeostasis and lipid metabolism pathways. Abbreviations: GLUTs, glucose transporters; AATs, amino acid transports; RTKs: receptor tyrosine kinases; IKKβ, IκB kinase β; AFP, alpha fetoprotein; PTEN, phosphatase and tensin homolog; SREBP1/2, sterol regulatory element-binding protein 1/2; PGC1α, PPARγ coactivator 1α; HIF1, hypoxia-inducible factor; TFEB, transcription factor EB.

mTOR signaling is a key regulator of many cellular processes including metabolism, proliferation and survival. As a consequence, the life cycles of numerous viruses are regulated by the mTOR signaling pathway (Ranadheera *et al.* 2018; Bossler *et al.* 2019). Notably, chronic HBV infection is one of the key factors in the development of HCC. Guo *et* *al.* demonstrated that HBV RNA transcription and subsequent DNA replication are inhibited by Akt, an upstream factor of mTORC1, in HBV-transfected cells (Guo *et al.* 2007b). These inhibitory effects appear to be mediated by mTORC1 activation because this inhibition can be reversed by rapamycin. In addition, PI3K, Akt and mTOR inhibition using high concentrations of drugs can promote HBV RNA transcription and DNA replication in vitro. Consistent with these findings, HBsAg expression in chronic hepatitis B is significantly upregulated compared with that in HBV-associated HCC (Wang M *et al.* 2013), which has a higher PI3K/Akt activity. However, low inhibitor concentrations of PI3K, Akt and mTOR mainly modulate posttranscriptional steps of HBV life cycle (Lin *et al.* 2020). The roles of HBV in the mTOR signaling pathway are summarized in Fig. 1.

### Role of HBsAg in the mTOR Signaling Pathway

The HBV entry process may activate the Akt/mTOR signaling pathway (Xiang and Wang 2018). However, short-term treatment with Akt inhibitors does not block HBV entry, suggesting that Akt activation induced by HBV infection is not essential for the viral entry process. Yet, it is not clear how the activation of the Akt/mTOR pathway is triggered. It is reported that uptake of various ligands or virus particles may activate the cellular Akt/mTOR pathways (Ji and Liu 2008; Eaton *et al.* 2014). This process needs to be examined in the future. HBV virions and noninfectious SVPs contain three glycosylated HBV surface antigens, S-, M-, and L-HBsAg (Mehta *et al.* 1997; Sigrid Schmitt 1999). HBV infection can induce the synthesis of a large quantity of viral proteins and lead to the accumulation of HBsAg in the ER lumen to trigger ER stress, thereby activating the PI3K/Akt/mTOR pathway (Choi *et al.* 2019). L-HBsAg has been shown to activate the PI3K/Akt/mTOR pathway to promote tumorigenesis (Liu *et al.* 2011). Consistent with this evidence, Teng *et* *al.* reported that wild type or mutant pre-S proteins can activate mTOR in Huh7 cells (Teng *et al.* 2011). It has also been reported that pre-S1 deletions lead to accumulation of L-HBsAg, thereby triggering ER stress to activate the mTOR signaling pathway (Choi *et al.* 2019). The pre-S mutant proteins can induce ER stress, leading to formation of ground glass hepatocytes (GGHs) (Wang *et al.* 2003). In addition, L-HBsAg and pre-S2 mutants activate the mTOR signaling pathway through induction of ER stress-dependent vascular endothelial growth factor (VEGF) A and Akt activation, resulting in aberrant glucose uptake and lactate production in tumorigenic processes. In accordance with this finding, the pre-S2 mutant proteins also trigger ER stress-mediated VEGF/Akt/mTOR and NF-κB/COX-2 signaling pathways to induce cell inflammation and transformation (Hung *et al.* 2004; Yang *et al.* 2009). Moreover, pre-S mutant-induced ER stress may also modulate the activity of SREBP1, ATP citrate lyase (ACLY), and fatty acid desaturase 2 (FADS2) (Teng *et al.* 2015b), major regulators of lipid metabolism, by activating the Akt/mTOR signaling pathway (Yang *et al.* 2009). Interestingly, activation of mTOR can inhibit HBsAg synthesis by promoting the interaction between histone deacetylase 1 (HDAC1) and the transcription factor YY1, which binds to the pre-S1 promoter (Teng *et al.* 2011). Disruption of HDAC1 eliminates the inhibitory effect of mTOR on pre-S transcription.

Collectively, the evidence indicates that wild type and mutant HBsAg accumulation can trigger ER stress and activate the Akt/mTOR signaling pathway. This process may promote tumorigenesis and is considered as an underlying mechanism of HBV-associated hepatocarcinogenesis. However, whether and how HBV may benefit from the HBsAg-induced ER stress remains elusive. Our recent approach using low doses of tunicamycin, a glycosylation inhibitor, showed that even artificially triggered ER-stress strongly promotes HBV replication and production by modulating viral assembly and release (unpublished data).

### Role of HBx in the mTOR Signaling Pathway

The HBx protein, the product of the smallest of the four overlapping open reading frames of the HBV genome, plays a vital role in HBV replication and the pathogenesis of HBV-associated HCC. HBx transfection in hepatoma cells increases the expression of mTOR (Wang P *et al.* 2013), and enhance the formation of autophagosomes and autolysosomes through the PI3K/Akt/mTOR signaling pathway (Wang P *et al.* 2013; Wang *et al.* 2014). HBX may also mediate the RAS/RAF/MAPK pathway activation, thereby inducing the mTOR signaling pathway by inhibiting the activity of TSC1/2 (Tarn *et al.* 2001; Chung *et al.* 2004). In addition, mTOR activation by HBx appears to be dependent on IκB kinase β (IKKβ), a major downstream kinase in the TNFα signaling pathway. IKKβ physically interacts with and phosphorylates TSC1 at Ser487 and Ser511, which results in TSC1 suppression and consequent mTOR activation (Lee *et al.* 2007). In addition, in an HBx transgenic (Tg) mouse model and HBV-associated HCC hepatocytes, HBx expression can regulate the IKKβ/mTOR/S6K1 signaling pathway (Yen *et al.* 2012). Disruption of IKKβ reverses HBx-mediated S6K1 activation, cell proliferation and VEGF production. Furthermore, HBx upregulates the expression of AFPR and AFP, which are early indicators of HBx-driven hepatocarcinogenesis, to activate the PI3K/Akt/mTOR signaling pathway (Zhu *et al.* 2015, 2017). Moreover, the HBx-induced antiapoptotic mechanism is essential for promoting the malignant transformation of hepatocytes via activation of the PI3K/Akt/mTOR signaling pathway (Wang X *et al.* 2019). The weakness of these studies is that the expression levels of HBx protein in transfected cells may be higher than that during HBV infection and replication and cause non-physiological effects.

The existing evidence suggests that HBx binds to and inactivates the transcription factor and tumor suppressor p53. Chung *et al.* reported that HBx disrupts p53-mediated transcription of PTEN, which is a negative regulator of the PI3K/Akt/mTOR signaling pathway (Chung *et al.* 2003). Consistent with this report, HBx can inhibit the expression (Lee and Rho 2000) or change the promoter binding site of p53 (Chan *et al.* 2013, 2016), which has been proven to inhibit the mTOR signaling pathway via activation of AMPK or TSC1/2 (Feng *et al.* 2005). Moreover, HBV proteins, including HBsAg and HBx, activate Wnt/β-catenin pathway (Cha *et al.* 2004; Lee *et al.* 2005; Daud *et al.* 2017) to inhibit GSK3 and TSC2, thereby promoting the mTOR signaling pathway (Inoki *et al.* 2006).

Altogether, the evidence indicates that HBx may indirectly activate the PI3K/Akt/mTOR signaling pathway, which plays a vital role in the development and progression of HBV-associated HCC. Though the reports mentioned above indicate an important role of HBx in the activation of mTOR signaling pathway, the findings are rather diverse and could not be integrated into a clearly unified concept. Future studies are required to find the connections between the events described in these studies.

### Role of HBV Infection in mTOR-Mediated Metabolism

The liver is an important organ for metabolic processes and plays key roles in glucose homeostasis and lipid metabolism. During HBV infection, many cellular signal transduction pathways are altered, including the PI3K/Akt/mTOR pathway (Teng *et al.* 2011, 2015a; Xu *et al.* 2013; Rawat and Bouchard 2015). The mTOR signaling pathway not only plays an essential role in coordinating anabolism and catabolism at the cellular level, but also has an important function in metabolic regulation in organisms (Saxton and Sabatini 2017).

With the discovery of a major bile salt carrier human sodium taurocholate cotransport peptide (hNTCP) as a functional receptor for HBV, the role of HBV in cellular metabolism has been placed at the forefront. Yan *et* *al.* showed that HBV pre-S1 may interfere with the physiological function of hNTCP to block bile acid uptake (Yan *et al.* 2014; Cheng *et al.* 2019). Decreased uptake of bile acid could promote compensatory bile acids synthesis and increase cholesterol supply to maintain homeostasis (Geier 2014; Patman 2014). In a transplant mouse model with HBV infection, an increased level of human cholesterol 7α-hydroxylase (CYP7A1), the rate-limiting enzyme that converts cholesterol to bile acids, was shown, consistent with decreased nuclear translocation of farnesoid X receptor (FXR, the positive transcription factor of SHP), and significantly reduced levels of SHP, the corepressor of hCYP7A1 transcription (Oehler *et al.* 2014).

HBV infection has been shown to regulate gluconeogenesis and aerobic oxidation of glucose. Park *et* *al.* showed that HBx acts as a positive regulator of gluconeogenesis (Shin *et al.* 2011). Increased HBx expression upregulates the expression of the key gluconeogenesis enzymes PEPCK and G6Pase and the production of hepatic glucose, leading to hyperglycemia and impaired glucose tolerance in HBx Tg mice. In addition, HBV pre-S2 mutants can induce aerobic oxidation of glucose by activating the mTOR signaling pathway (Teng *et al.* 2015a). HBV infection stimulates the expression of G6PD, the first and rate-limiting enzyme of the pentose phosphate pathway (PPP), through HBx-mediated nuclear factor erythroid 2-related factor 2 (Nrf2) activation (Liu B *et al.* 2015).

In addition, mTORC1 activation stimulates *de* *novo* lipogenesis by interacting with PPARγ (Li *et al.* 2014) and regulating the activity of SREBP1, two master regulators of lipid metabolism in hepatocytes (Peterson *et al.* 2011). It has been reported that HBx expression leads to lipid accumulation in hepatocytes mediated by SREBP1 and PPARγ (Kim *et al.* 2007). A number of studies have consistently demonstrated that HBV infection has an impact on lipid metabolism. Fatty acid binding protein 5 (Fabp5), acyl-CoA binding protein (ACBP), SREBP2, ACLY, and fatty acid synthase (FAS), which are involved in fatty acid metabolism and synthesis, are strongly upregulated in HBx Tg mice (Yang *et al.* 2008). HBV is an enveloped virus, and its successful assembly and secretion depend on the biogenesis of lipids and the formation of lipid membranes. HBx mediates the transcription of SREBP1, FAS, and PPARγ by activating lipogenesis induced by the nuclear receptor LXRα in HBV-associated HCC (Kim *et al.* 2008; Na *et al.* 2009). Furthermore, pre-S2 mutant-mediated induction of mTOR can activate SREBP1/ACLY/FAS, thereby inducing lipid accumulation (Teng *et al.* 2015b). On the other hand, HBV infection is associated with reduced prevalence of fatty liver, hypertriglyceridemia and metabolic syndrome in patients (Wong *et al.* 2012). This observation may have different interpretations and needs to be analyzed further.

Taken together, HBV infection interferes with hepatic metabolism pathways, including glucose homeostasis and bile acid and lipid metabolism pathways, and eventually leads to metabolic disorders. Alteration of these metabolic pathways may also contribute to the pathological process of HBV-associated HCC. On the other hand, viral infections require active metabolisms to supply energy and building blocks (Claus and Liebert 2014). HBV life cycle is also strongly dependent on host cell metabolism and its regulation (also see Conclusion). There are still many aspects in this direction for future research, for example, how different metabolic pathways and metabolites influence HBV replication via mTOR signaling pathways. Therefore, studies on the metabolic pathways during HBV infection could provide new insights and therapeutic approaches.

## mTOR Inhibits HBV Replication

The activation of mTOR by HBV infection can enhance cellular metabolism, autophagy, and immune responses to reduce the damage to host cells. Although the PI3K/Akt/mTOR pathway benefits most viruses, the PI3K/Akt/mTOR pathway has been shown to reduce HBV replication in some experimental conditions and may be partly responsible for low or absent HBV replication in transformed and tumor cells. Specifically, triggering the PI3K/Akt/mTOR signaling pathway directly inhibits the transcription of HBV 3.5-kb and 2.4-kb RNA and HBV replication in hepatoma cells (Guo *et al.* 2007b). In addition, mTOR can inhibit HBV replication by preventing the recruitment of the YY1-HDAC1 complex to the pre-S1 promoter (Teng *et al.* 2011). However, the interaction between HBV replication and mTOR-regulated cellular processes is far more complex.

**Fig. 2:**
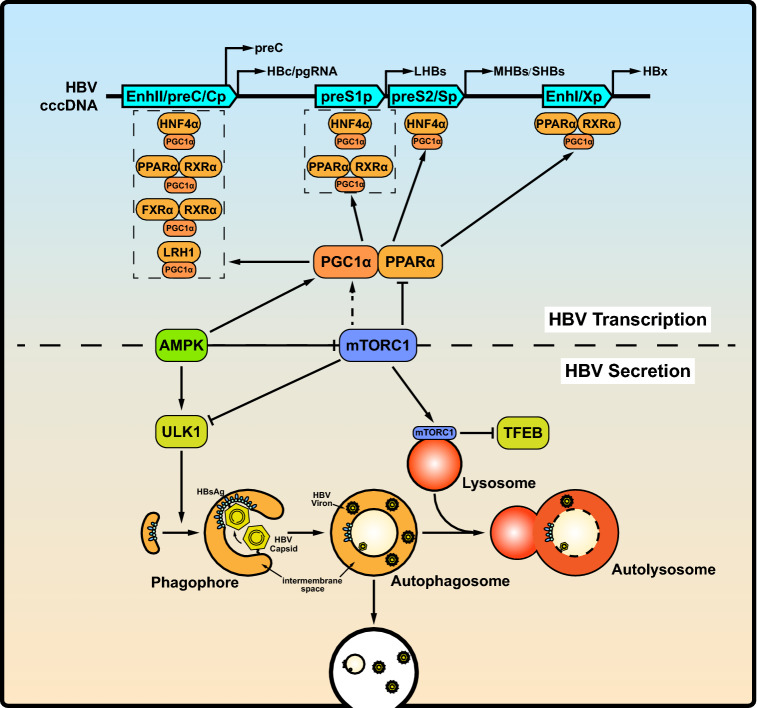
mTOR-mediated host responses regulate HBV RNA transcription, assembly and secretion of virions and subviral particles. Top: mTOR and AMPK, two major metabolic sensors, control the activity of PPARα and PGC1α to regulate HBV transcription through modulating HBV enhancers and promoters. Bottom: mTOR inhibits both the early (autophagosome formation) and late (lysosomal activity) phases of autophagy. HBsAg utilizes early autophagic structures for envelope formation. HBV virions and subviral particles are degraded during the late phase of autophagy. Abbreviations: PPARα, peroxisome proliferator-activated receptor alfa; PGC1α, PPARγ coactivator 1α; FXRα, farnesoid X receptorα; RXR, retinoid X receptor alpha; LRH1, liver receptor homolog 1; HNF4α, hepatocyte nuclear factor 4α; TFEB, transcription factor EB.

The mTOR kinase complex negatively regulates autophagy initiation and its inhibition activates the ULK kinase complex association with the PIK3C3/BECN1/ATG14 complex, leading to the formation of autophagosomes. Then, autophagosomes fuse with lysosomes to degrade cytoplasmic cargo (Fig. 2, bottom), whose activity also negatively regulated by the mTOR kinase (Lin *et al.* 2019c; Wang *et al.* 2021). Studies have found that autophagosome formation is essential for efficient HBV replication in different cell and animal model (Sir *et al.* 2010; Li *et al.* 2011; Tian *et al.* 2011; Lin *et al.* 2019a; Lin *et al.* 2019b; Lin *et al.* 2019c; Wang J *et* *al.* 2019; Wang *et al.* 2020a; Wang *et al.* 2020b). In contrast, autophagy inhibition can prevent HBV replication both *in vivo* and *in* *vitro*.

Recent studies demonstrated that HBV replication and assembly can be promoted by initiating the early phase of autophagy. However, a great part of HBV antigens and virions is degraded during the late phase of autophagy. Sir *et* *al.* showed that HBV DNA replication is markedly reduced by the PtdIns3K inhibitor 3-methyladenine (3-MA) or siRNA that targets the *PtdIns3K* or *ATG7* gene to disrupt the initiation of autophagy (Sir *et al.* 2010). The noncoding microRNA-99 family targets Akt and mTOR to induce autophagy, thereby promoting HBV replication (Lin *et al.* 2017). Consistent with these findings, our previous study found that treatment with glucosamine (Lin *et al.* 2019c) or disruption of *O*-GlcNAcylation by OSMI-1 or siRNA that targets OGT greatly enhances HBV replication by initiating the early phase of autophagy by inhibiting mTOR signaling pathway (Wang *et al.* 2020b). Moreover, autophagy is initiated by inhibition of the Akt/mTOR signaling pathway at low glucose concentrations, with enhanced HBV replication at the same time (Wang *et al.* 2020a). Our recent study demonstrated that HBV inhibited lysosomal activity is restored to enhance HBV antigens degradation by inhibiting Akt/mTOR signaling after silencing CCDC88A in hepatoma cells with HBV replication (Wang *et al.* 2021). Some studies have reported that certain drugs, such as rapamycin (an mTOR inhibitor), cisplatin and dexamethasone, induce the initiation of autophagy and promote HBV replication by inhibiting the mTOR signaling pathway (Huang *et al.* 2014; He *et al.* 2018; Chen *et al.* 2019).

The critical role of autophagy in HBV replication has not only been demonstrated in HBV cell culture models but also *in* *vivo*. Tian *et* *al.* showed that the serum HBV DNA levels are significantly reduced and the HBV DNA replication intermediates were almost undetectable in HBV transgenic mice if the *Atg5* gene is specifically knocked out in the liver (Tian *et al.* 2011). Our recent study illustrated that glucosamine increases HBV replication by blocking mTOR signaling via a feedback mechanism in a HBV mouse model (Lin *et al.* 2019c).

To maintain effective autophagic flux, autophagosome formation is linked with autophagosomal-lysosomal fusion and lysosomal degradation activity. Although mTOR regulates both early (autophagosome formation) and late (lysosomal activity) phase of autophagy, HBV assembly and release through enhanced autophagy are still beneficial for the production HBV particles despite the degradation by lysosomes. Recent findings also showed that HBV replication can interfere with the lysosomal functions and thereby evade the autophagic degradation process (Xie *et al.* 2016; Lin *et al.* 2019a, 2019b). A part of autophagosomes may not fuse with lysosomes for degradation, but rather release HBV proteins and virions through other pathways, such as fusing with MVBs (Wang *et al.* 2021) or exosomes as indicated by our unpublished work.

As mTOR pathway integrates many different signals, indirect crosstalk with some other major pathways may occur, thereby regulating downstream processes and HBV replication. For example, type I interferon signaling has been shown to crosstalk with Akt/mTOR signaling (Uddin *et al.* 1995; Lekmine *et al.* 2004). Our unpublished data revealed that type I interferon may modulate autophagy and subsequently HBV replication via Akt/mTOR pathway.

## Conclusions and Perspectives

In this review, we have summarized recent progress from studies on the interaction between the cellular mTOR signaling pathway and HBV replication. mTOR first attracted the attention of researchers because it may play important roles in the development of HBV-associated HCC. The mTOR inhibitors are able to suppress cell growth *in* *vitro* and *in* *vivo*. Viral infections often alter host cellular metabolisms through regulating various cellular factors and pathways, including the PI3K/Akt/mTOR signaling pathway. The mTOR signaling pathway is essential in hepatocyte glucose and lipid metabolism, which are two important biological processes for efficient HBV replication. Additionally, mTOR negatively regulates autophagosome formation by inactivating the ULK kinase complex bound to the PIK3C3/BECN1/ATG14 complex and thereby reducing HBV assembly and release through autophagy. Up to date, the mTOR-mediated signaling pathway has been shown to be essential for HBV replication and pathogenesis in cell culture models. However, only few studies have been conducted in human tissues or animal models and need to be continued in the future. In addition, there are limitations in studying the cross-regulation between HBV infection and mTOR signaling in human tissues or animal models. The development of new technologies in molecular and cell biology will help to better understand the cross-regulation between HBV infection and mTOR signaling. In the future, this may help identify new therapeutic targeting.

Different concentrations of PI3K, Akt and mTOR inhibitors have different effects on HBV transcription and replication. Guo *et* *al.* showed that treatment of HBV-expressing HepG2.2.15 cells with high concentrations of inhibitors of PI3K, Akt, and mTOR (10, 5, and 10 μmol/L, respectively) increased the transcription of 3.5-kb and 2.4-kb viral RNA as well as the replication of HBV DNA (Guo *et al.* 2007b). However, some other studies including ours showed that low concentrations (≤ 1 μmol/L) of the inhibitors significantly enhanced HBV replication through enhancing autophagy instead of HBV transcription (Huang *et al.* 2014; Lin *et al.* 2020). There are many binding sites of ubiquitous and hepatocyte-enriched transcription factors in the promoter and enhancer regions of the HBV genome, including that for the transcription factors PGC1α and the PPARα/retinoid X receptor alpha (RXRα) complex. Previously evidences suggested that mTOR could regulate the activity of PGC1α and PPAR *in* *vitro* or *in* *vivo* (Raney *et al.* 1997; Shlomai *et al.* 2006; Shlomai and Shaul 2009; Reese *et al.* 2011; Weng *et al.* 2014), which play essential roles in HBV transcription. Moreover, PGC1α can be recruited by nuclear receptors (Fig. 2, top), including hepatocyte nuclear factor 4α (HNF4α), the PPARα/RXRα heterodimer, the FXRα/RXRα heterodimer, and liver receptor homolog 1 (LRH1), which positively regulate HBV pgRNA synthesis to enhance HBV replication (Reese *et al.* 2011; Weng *et al.* 2014). Therefore, high-concentrations of PI3K, Akt, and mTOR inhibitors may have a stronger regulatory effect on these transcription factors, while low-concentrations of these inhibitors mainly regulate the mTOR-induced autophagy pathway and have lower regulatory effects on these transcription factors. However, this hypothesis needs to be tested in future experimental approaches. It is likely that these nutrient-regulated transcription factors on HBV transcription also are regulated by HBV activated mTORC1.

Recently, increasing evidence has indicated that the mTOR signaling pathway plays multiple roles in immunity (Weichhart *et al.* 2015). The interaction of S6K and STING can regulate the activation of IRF3 to induce innate immunity (Wang *et al.* 2016). The mTOR signaling pathway can regulate c-Fos expression (He *et al.* 2015), and IFNα/β production (Cao *et al.* 2008). Besides, Delgoffe *et* *al.* reported that naïve T cells fail to differentiate into Th1, and Th17 cells in mTOR or Rheb gene knockout mice (Delgoffe *et al.* 2009, 2011). In addition, rapamycin at a high dose can attenuate CD8^+^ T cell responses (Pearce *et al.* 2009), and exerts immunostimulatory effects on memory CD8^+^ T cell differentiation (Araki *et al.* 2010). Thus, mTOR is involved in the regulation of both innate and adaptive immunity. It is tempting to speculate that mTOR-mediated innate or adaptive immunity may contribute to control HBV infection. Importantly, further understanding of the immunity and mTOR signaling may provide novel targets for regulating immunity and treating HBV-related disease.

Despite recent advances in drug discovery and development, effective antiviral drugs against HBV are still very limited, especially for the cure of chronic HBV infection. Thus, the development of effective, well-tolerated and affordable antiviral treatments is necessary and urgent. The HBV-induced abnormalities in the expression of PI3K/Akt/mTOR and its downstream regulators and alteration in host metabolism make mTOR a potential target for drug development.
